# Counter-regulatory responses to postprandial hypoglycaemia in patients with post-bariatric hypoglycaemia vs surgical and non-surgical control individuals

**DOI:** 10.1007/s00125-022-05861-9

**Published:** 2023-01-17

**Authors:** Afroditi Tripyla, David Herzig, Gemma Reverter-Branchat, Jacopo Pavan, Michele Schiavon, Philippe J. Eugster, Eric Grouzmann, Christos T. Nakas, Valérie Sauvinet, Laure Meiller, Joerg Zehetner, Daniel Giachino, Philipp Nett, Joanna Gawinecka, Simone Del Favero, Andreas Thomas, Mario Thevis, Chiara Dalla Man, Lia Bally

**Affiliations:** 1 Department of Diabetes, Endocrinology, Nutritional Medicine and Metabolism, Inselspital, Bern University Hospital, University of Bern, Bern, Switzerland; 2 Department of Information Engineering, University of Padova, Padova, Italy; 3 Laboratory of Catecholamines and Peptides, Service of Clinical Pharmacology, Lausanne University Hospital and University of Lausanne, Lausanne, Switzerland; 4 School of Agricultural Sciences, Laboratory of Biometry, University of Thessaly, Volos, Greece; 5 University Institute of Clinical Chemistry, Inselspital, Bern University Hospital, University of Bern, Bern, Switzerland; 6 Centre de Recherche Nutrition Humaine Rhône-Alpes, Univ-Lyon, Inserm, INRAe, Claude Bernard Lyon1 University, Hospices Civils de Lyon, Centre Hospitalier Lyon Sud, Pierre Bénite, France; 7 Department of Visceral Surgery, Hirslanden Clinic Beau-Site, Bern, Switzerland; 8 Department of Visceral Surgery, Lindenhofspital, Bern, Switzerland; 9 Department of Visceral Surgery and Medicine, Inselspital, Bern University Hospital, University of Bern, Bern, Switzerland; 10 Institute of Clinical Chemistry, University Hospital Zurich, University of Zurich, Zurich, Switzerland; 11 Institute of Biochemistry / Center for Preventive Doping Research, German Sport University Cologne, Cologne, Germany

**Keywords:** Gastric bypass, Glucagon, Glucose metabolism, Sleeve gastrectomy, Weight

## Abstract

**Aims/hypothesis:**

Post-bariatric hypoglycaemia is an increasingly recognised complication of bariatric surgery, manifesting particularly after Roux-en-Y gastric bypass. While hyperinsulinaemia is an established pathophysiological feature, the role of counter-regulation remains unclear. We aimed to assess counter-regulatory hormones and glucose fluxes during insulin-induced postprandial hypoglycaemia in patients with post-bariatric hypoglycaemia after Roux-en-Y gastric bypass vs surgical and non-surgical control individuals.

**Methods:**

In this case–control study, 32 adults belonging to four groups with comparable age, sex and BMI (patients with post-bariatric hypoglycaemia, Roux-en-Y gastric bypass, sleeve gastrectomy and non-surgical control individuals) underwent a postprandial hypoglycaemic clamp in our clinical research unit to reach the glycaemic target of 2.5 mmol/l 150–170 min after ingesting 15 g of glucose. Glucose fluxes were assessed during the postprandial and hypoglycaemic period using a dual-tracer approach. The primary outcome was the incremental AUC of glucagon during hypoglycaemia. Catecholamines, cortisol, growth hormone, pancreatic polypeptide and endogenous glucose production were also analysed during hypoglycaemia.

**Results:**

The rate of glucose appearance after oral administration, as well as the rates of total glucose appearance and glucose disappearance, were higher in both Roux-en-Y gastric bypass groups vs the non-surgical control group in the early postprandial period (all *p*<0.05). During hypoglycaemia, glucagon exposure was significantly lower in all surgical groups vs the non-surgical control group (all *p*<0.01). Pancreatic polypeptide levels were significantly lower in patients with post-bariatric hypoglycaemia vs the non-surgical control group (median [IQR]: 24.7 [10.9, 38.7] pmol/l vs 238.7 [186.3, 288.9] pmol/l) (*p*=0.005). Other hormonal responses to hypoglycaemia and endogenous glucose production did not significantly differ between the groups.

**Conclusions/interpretation:**

The glucagon response to insulin-induced postprandial hypoglycaemia is lower in post-bariatric surgery individuals compared with non-surgical control individuals, irrespective of the surgical modality. No significant differences were found between patients with post-bariatric hypoglycaemia and surgical control individuals, suggesting that impaired counter-regulation is not a root cause of post-bariatric hypoglycaemia.

**Trial registration:**

ClinicalTrials.gov NCT04334161

**Graphical abstract:**

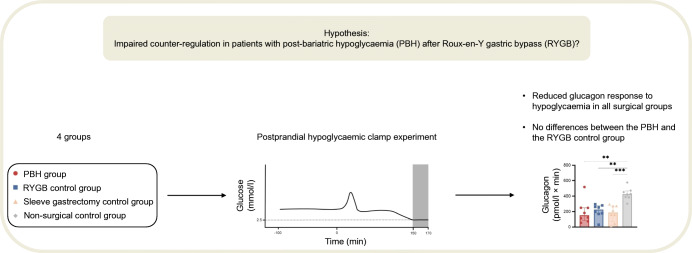

**Supplementary Information:**

The online version contains peer-reviewed but unedited supplementary material available at 10.1007/s00125-022-05861-9.

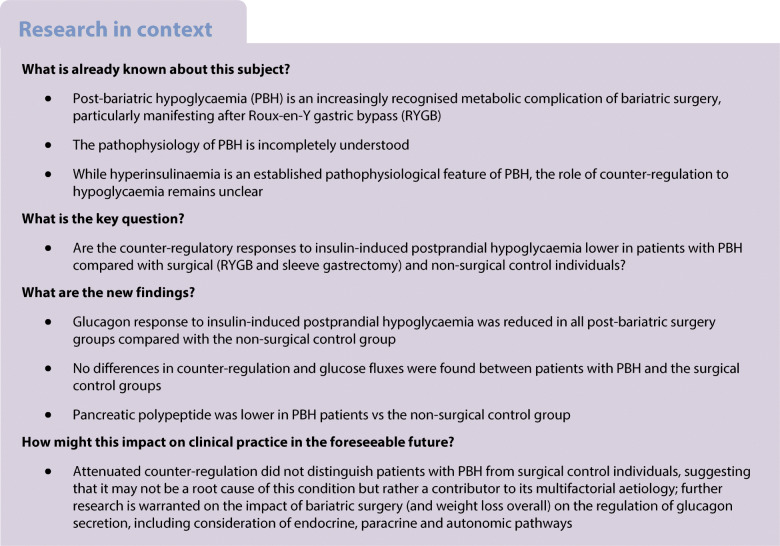



## Introduction

Roux-en-Y gastric bypass (RYGB) and sleeve gastrectomy (SG) are the two most commonly performed bariatric surgery procedures and currently remain the most potent anti-obesity treatment modalities [1, 2]. Despite their well-established positive effects on obesity-related comorbidities [3–5], post-bariatric hypoglycaemia (PBH) is an increasingly recognised late metabolic complication, particularly manifesting after RYGB [6]. Although the reported incidence ranges widely because of the heterogenic diagnostic criteria used [7–9], a recent study suggested that PBH may affect up to 30% of post-RYGB individuals [10]. PBH typically occurs after meals with high glycaemic impact as a consequence of inappropriate insulin exposure. Although heightened insulinotropic factors, particularly glucagon-like peptide-1 (GLP-1), have been suggested as key pathophysiological features [11], the considerable phenotypic variability among patients with PBH and the high prevalence of asymptomatic hypoglycaemic episodes [12, 13] suggest that impaired counter-regulation may contribute to the pathophysiology of PBH. Additionally, post-RYGB glucagon exposure during a provocative test has been found to negatively correlate with the time spent in hypoglycaemia [14]. In another study, RYGB patients experiencing hypoglycaemia during a provocative test showed inadequate counter-regulatory hormonal responses compared with non-hypoglycaemic counterparts [15]. However, a proper evaluation of counter-regulatory hormones, including their effects on glucose fluxes during hypoglycaemia, requires a standardised hypoglycaemic clamp experiment exposing well-identified affected and non-affected individuals to identical hypoglycaemic levels.

The objective of this study was to assess the counter-regulatory hormones and glucose fluxes to standardised insulin-induced postprandial hypoglycaemia in patients with confirmed PBH after RYGB compared with the responses of control groups (individuals without PBH after RYGB and SG procedures, and non-surgical individuals not undergoing surgery). Due to its central and immediate role in counter-regulation [16], glucagon response was defined as the primary outcome. We hypothesised that glucagon response to postprandial hypoglycaemia is diminished in patients with PBH when compared with surgical and non-surgical control individuals.

## Methods

### Study design and population

This observational case–control study was conducted at the Inselspital, Bern University Hospital in Switzerland between October 2020 and July 2021. Thirty-two adults belonging to four groups (*n*=8 each) were recruited: patients with PBH after RYGB; individuals without PBH after RYGB; individuals without PBH after SG; and a non-surgical control group (CN). Individuals belonging to the control groups were selected based on best-fit alignment to the PBH group in terms of age, sex and BMI. CN participants were recruited using advertisements on the hospital website.

Individuals were eligible for inclusion in the surgical groups if they had been operated on ≥1 year ago. The PBH group was identified from a pool of patients with PBH who reported neuroglycopenic symptoms and tested positive in a routine-care diagnostic work-up (plasma and sensor glucose <3.0 mmol/l [12, 17] during a provocative test and masked continuous glucose monitoring). Further details of the PBH group are given in electronic supplementary material (ESM) Table 1. Participants in the RYGB, SG and CN groups needed to demonstrate unremarkable capillary glucose records (≥3.9 mmol/l) for 2.5 h following a standardised breakfast (2290.6 kJ [548 kcal]; 70 g carbohydrates, 23 g fat, 14 g protein) and no evidence of postprandial hypoglycaemia in their medical history/records. Exclusion criteria across all groups were medical conditions including present or past history of diabetes (defined as HbA_1c_ ≥48 mmol/mol [6.5%]), renal dysfunction, liver or gastrointestinal disorders, anaemia, pregnancy, breastfeeding and weight fluctuations (≥5% within the prior 3 months). Any pharmacological agents interfering with glucose regulation were stopped prior to study-related procedures.

The study was conducted in accordance with the World Medical Association Declaration of Helsinki (as revised in 2008), the principles of Good Clinical Practice and the local legally applicable requirements. The Ethics Committee Bern approved the study (2020-00400) and participants provided written informed consent prior to study-related procedures.

### Experimental procedures

Following enrolment, participants’ body weight, height and body composition (Inbody 770; Inbody Co., Seoul, South Korea) were measured.

Participants were instructed to avoid physical activity, alcohol and caffeine for 48 h before the study experiment. After an overnight fast, they arrived at the clinical research unit and i.v. catheters were inserted into each forearm, one for blood withdrawal and one for the infusion of insulin, glucose and [6,6-^2^H_2_]glucose. After obtaining blood samples for background isotopic glucose enrichments, a primed (3 mg/kg) continuous (30 μg kg^−1^ min^−1^) [6,6-^2^H_2_]glucose (Cambridge Isotope Laboratories, Tewksbury, USA) infusion was started 100 min before the participants ingested 15 g of glucose (0 min [t0]) (Roquette Frères, Lestrem, France). Glucose was labelled with 1.5% [U-^13^C]glucose (Cambridge Isotope Laboratories), dissolved in 200 ml water and was ingested within 5 min with the participants sitting in an upright sitting position. A continuous insulin aspart infusion (diluted in NaCl 154 mmol/l, 0.0623 U kg^−1^ h^−1^; Novo Nordisk A/S, Bagsvaerd, Denmark) was initiated at 90 min (t90). Using a proportional integrative derivative control algorithm [18], the glucose infusion rate (GIR) (Glucose Bioren 20%; Sintetica, Mendrisio, Switzerland) was modulated to achieve a standardised glycaemic decrease. The glycaemic target of 2.5 mmol/l was reached at 150 min (t150) and was maintained for 20 min. At 170 min (t170), the insulin aspart infusion was stopped and the GIR was increased to restore euglycaemia. In five experiments (two with delayed achievement of the hypoglycaemic plateau and three with low blood flow challenging blood sampling), the 20 min hypoglycaemic period was right-shifted. In the data analysis, the hypoglycaemic periods of all participants were aligned and the terms ‘hypoglycaemic period’ and ‘t150–t170’ are used interchangeably in the manuscript.

Blood glucose was initially measured every 15 min, and from 75 min (t75) every 5 min using the Accu-Chek Inform II meter (Roche Diagnostics, Mannheim, Germany). Participants were blinded to their glucose levels. Blood samples for endogenous insulin, C-peptide, insulin aspart, glucagon, adrenaline (epinephrine), noradrenaline (norepinephrine), cortisol, growth hormone (GH), pancreatic polypeptide (PP), GLP-1 and isotopic glucose enrichments were collected at pre-defined time points.

ECG recordings were performed during the entire experiment using a three-channel ECG with a Lifecard CF digital holter recorder (Spacelabs Healthcare, Hertford, UK). Systolic and diastolic BP was also measured at pre-defined time points (Shenzhen Mindray Bio-Medical Electronics Co., Shenzhen, China).

Hypoglycaemic symptoms were assessed using the Edinburgh Hypoglycaemia Symptom Scale [19], in which four autonomic symptoms (sweating, palpitations, tremor, hunger), five neuroglycopenic symptoms (confusion, dizziness, odd behaviour, speech difficulties, incoordination) and two general malaise symptoms (headache and nausea) were rated on a Likert scale from 1 (absent) to 7 (severe).

The experimental flow and the timing of the examined variables are shown in ESM Fig. 1 and ESM Table 2.

### Assays

Samples for glucagon (collected in EDTA combined with aprotinin), insulin aspart (collected in EDTA), catecholamines and isotopic glucose enrichments (both collected in heparin), and PP and GLP-1 (collected in pre-chilled heparin combined with a protease inhibitor cocktail [20]) were immediately centrifuged (2500 *g* at 4°C for 10 min, apart from isotopic glucose enrichments [3645 *g* at 4°C for 10 min]). Samples for endogenous insulin, C-peptide, cortisol and GH (collected in serum) were kept at room temperature for 30 min before centrifugation. All samples were stored at −80°C until assayed, except for isotopic glucose enrichments (−20°C).

Glucagon was measured using a modified, sequential sandwich immunoassay (no. 10-1271-01; Mercodia, Uppsala, Sweden) with an additional washing step to reduce non-specific binding of glucagon-related molecules [21]. Immunometric assays were also used to quantify endogenous insulin (Elecsys, 0% cross-reactivity with insulin aspart [22]; Roche Diagnostics), C-peptide, GH (both Immulite 2000; Siemens Healthcare Diagnostics, Tarrytown, USA), cortisol (Elecsys Cortisol II; Roche Diagnostics) and GLP-1 (GLP-1 7-36 and 7-37; Immuno-Biological Laboratories Co., Fujioka, Japan). Insulin aspart [23], catecholamines [24], PP1-36 and PP3-36 [20] were quantified using LC-MS/MS. The sum of the two PP fragments is reported as total PP. Plasma [6,6-^2^H_2_]glucose was measured by gas chromatography–mass spectrometry in positive chemical ionisation (GC-CI/MS; GC 6890, MS 5973; Agilent, Massy, France) and plasma [U-^13^C]glucose by gas chromatography-combustion-isotope ratio mass spectrometry (GC/C/IRMS; GC Trace-Isolink coupled to the Delta V Advantage via the ConflowIV interface; Thermo Scientific, Bremen, Germany) [25]. Precision details of the used assays are shown in ESM Table 3.

### Glucose fluxes

The rate of glucose appearance after oral administration (*R*_a_O), rate of endogenous glucose production (EGP), rate of total glucose appearance (*R*_a_tot) and rate of glucose disappearance (*R*_d_) were computed from plasma [6,6-^2^H_2_]glucose and [U-^13^C]glucose enrichments as well as the total glucose concentration in plasma, using a dual-tracer approach and the one-compartment Steele’s equation for the non-steady state [26, 27]. Further information is provided in ESM Methods.

### Calculations

#### Postprandial period

To characterise group-specific differences, metrics of glucose–insulin homeostasis were calculated in the fasting and the postprandial state. Insulin sensitivity and beta cell function in the fasting state were estimated with the HOMA2 calculator (version 2.2.3; Diabetes Trials Unit, Oxford, UK) [28] using fasting blood glucose as well as endogenous insulin and C-peptide levels, respectively. In the early postprandial period, peak blood glucose, peak endogenous insulin levels and the respective time-to-peak were assessed. Additionally, the peak insulin secretion rate (ISR) (ISR computed using ISEC software, version 3.4a; Metabolic Modelling Group, London, UK [29]) was also considered. Insulin metrics, such as insulin secretion (shown as AUC of the ISR using the trapezoidal rule), insulin sensitivity (estimated based on the *R*_d_ adjusted for blood glucose and endogenous insulin levels: *R*_d_/glucose/insulin, calculated as ml min^−1^ kg^−1^ per nmol/l, as previously described [30, 31]) and total insulin clearance (AUC ISR/AUC endogenous insulin), were also assessed during the entire postprandial period (t0–t85).

Early postprandial glucose fluxes were characterised by calculating the AUC_0-15_ of *R*_a_O, *R*_a_tot, and *R*_d_ using the trapezoidal rule and by estimating the EGP suppression at t15 as relative difference to t0. The percentage of orally administered glucose reaching the systemic circulation during the entire postprandial period was computed as the AUC_0-85_ of *R*_a_O divided by the oral glucose load.

#### Hormonal response during the hypoglycaemic period

The primary outcome of this study was the incremental AUC (iAUC) of glucagon during hypoglycaemia (t150–t170), assessed using the trapezoidal rule and in relation to t85 (before insulin initiation). The iAUCs and the mean levels of catecholamines, cortisol and GH were calculated during t150–t170. The levels of PP and GLP-1 were also assessed at t160.

#### Metabolic response during the hypoglycaemic period

The mean and AUC of glucose fluxes and GIR (calculated using the trapezoidal rule) were assessed during t150–t170. Insulin sensitivity during hypoglycaemia was calculated based on the *R*_d_ adjusted for blood glucose and total insulin levels (endogenous insulin and insulin aspart), as described above. The post-hepatic insulin clearance was estimated using a linear one-compartment model for insulin distribution [32] (for further details, see ESM Methods).

#### Cardiocirculatory response during the hypoglycaemic period

ECG recordings were uploaded to the Sentinel software (version 11.5.1.12779; Spacelabs Healthcare), from which the heart rate was exported. The mean heart rate during t150–t170, the systolic and diastolic BP at t165 and absolute differences to baseline (t0) were calculated.

#### Hypoglycaemic symptoms

The total, autonomic, neuroglycopenic and general malaise symptom sum scores were calculated at t165. Of note, the symptom sum score ranges are as follows: total sum score, 11–77; autonomic sum score, 4–28; neuroglycopenic sum score, 5–35; and general malaise sum score, 2–14.

### Statistical analysis

Due to the lack of pre-existing literature addressing the primary outcome in the target population, no formal sample size was calculated and we aimed for 32 participants (eight in each group) with complete data.

Group differences were assessed using one-way ANOVA or the Kruskal–Wallis test, if after logarithmic transformation the ANOVA assumptions were still not met. Post hoc comparisons between all groups were performed using the Tukey’s Honest Significant Differences test or the Mann–Whitney test, respectively. A *p* value of <0.05 was considered statistically significant. All reported pairwise *p* values relate to comparisons with CN. For other statistically significant pairwise comparisons, the respective *p* values are reported. For variables with only two sampling time points (such as BP, measured at t0 and t165), the change over time was assessed using paired *t* tests or the Wilcoxon signed-rank test. Data are presented as median (IQR). Statistical analyses were performed using R (version 4.0.1; The R Foundation for Statistical Computing, Vienna, Austria) and data visualisation was performed using Prism (version 9.2.0; GraphPad Software, San Diego, USA) and Adobe Photoshop (version 21.0.3; Adobe, San Jose, USA).

## Results

### Study participants and baseline metabolic assessment

Eighty-seven individuals were invited to participate, of which 45 accepted and 35 were recruited. Ten individuals foreseen as control participants (eight RYGB, one SG and one CN) experienced hypoglycaemia (<3.9 mmol/l) after the standardised breakfast and were excluded. Three participants had incomplete data during the experiment and were not included in the analysis (for further details, see ESM Fig. 2).

The four groups had similar age, women/men ratio, BMI, body composition and HbA_1c_ levels (Table 1). Time since surgery and weight loss were also comparable between the surgical groups. The following treatments were stopped before study-related procedures: beta blocker in one SG and one CN group member; beta adrenergic bronchodilator in one CN group member; and acarbose in one PBH group member.

**Table 1:** Baseline characteristics of study groups

Characteristic	PBH group (*n*=8)	RYGB control group (*n*=8)	SG control group (*n*=8)	CN (*n*=8)	*p* value
Age, years	47.5 (30.6 – 48.9)	45.2 (37.1 – 52.1)	49.2 (31.2 – 52.3)	49.0 (31.4 – 54.2)	0.948
Sex, women:men	7:1	7:1	7:1	7:1	NA
BMI, kg/m^2^	29.7 (24.1 – 32.0)	27.5 (24.2 – 31.0)	30.2 (24.8 – 33.4)	26.9 (24.8 – 31.6)	0.865
Weight, kg	78.7 (71.2 – 81.8)	75.6 (67.5 – 83.2)	79.7 (74.5 – 92.0)	78.4 (67.4 – 87.0)	0.726
Fat mass, kg	26.0 (18.4 – 32.4)	24.0 (20.7 – 31.9)	31.6 (21.8 – 36.9)	22.7 (17.9 – 39.5)	0.830
Fat-free mass, kg	47.5 (37.9 – 53.5)	48.6 (45.7 – 55.6)	51.5 (47.8 – 59.4)	46.8 (43.3 – 51.7)	0.540
HbA_1c_, mmol/mol	31 (30 – 33)	31 (30 – 32)	34 (32 – 36)	32 (28 – 32)	0.197
HbA_1c_, %	5.0 (4.9 – 5.2)	5.0 (4.9 – 5.1)	5.3 (5.1 – 5.4)	5.1 (4.7 – 5.1)	0.197
Time since surgery, years	6.2 (5.4 – 6.9)	5.6 (3.1 – 6.8)	5.1 (3.2 – 5.8)	NA	0.362
Pre-operative BMI, kg/m^2^	41.8 (41.0 – 43.8)	39.5 (38.3 – 46.2)	39.2 (39.0 – 42.2)	NA	0.698
Pre-operative weight, kg	113.3 (111.3 – 119.0)	114.0 (99.7 – 134.9)	118.3 (109.3 – 126.5)	NA	0.940
Total weight loss, %	31.4 (25.6 – 39.0)	32.6 (29.2 – 40.0)	28.7 (20.2 – 36.6)	NA	0.569
Fasting blood glucose, mmol/l	4.9 (4.7 – 5.0)	5.1 (5.0 – 5.1)	4.9 (4.7 – 5.1)	4.9 (4.5 – 5.2)	0.538
Fasting endogenous insulin, pmol/l	32.1 (26.1 – 37.7)	36.6 (23.7 – 41.6)	31.8 (27.2 – 35.2)	39.0 (32.0 – 45.4)	0.628
Fasting C-peptide, nmol/l	0.5 (0.4 – 0.6)	0.6 (0.5 – 0.7)	0.4 (0.4 – 0.5)	0.4 (0.4 – 0.5)	0.234
HOMA2 %S	145.6 (125.4 – 180.8)	124.0 (113.7 – 196.7)	148.3 (134.0 – 173.1)	120.5 (106.6 – 150.8)	0.584
HOMA-IR^a^	0.7 (0.6 – 0.8)	0.8 (0.5 – 0.9)	0.7 (0.6 – 0.7)	0.8 (0.7 – 1.0)	0.655
HOMA2 %B	108.8 (96.8 – 116.4)	102.9 (94.0 – 117.2)	99.0 (90.6 – 107.8)	88.6 (86.6 – 102.7)	0.712
Fasting glucagon, pmol/l	3.4 (2.1 – 5.6)	4.5 (2.6 – 5.9)	3.8 (2.4 – 6.7)	4.9 (4.1 – 6.7)	0.783
Fasting adrenaline, nmol/l	0.08 (0.05 – 0.1)	0.11 (0.07 – 0.23)	0.08 (0.04 – 0.17)	0.1 (0.04 – 0.15)	0.938
Fasting noradrenaline, nmol/l	1.5 (1.4 – 2.1)	1.2 (1.1 – 1.4)	1.7 (1.5 – 2.4)	2.0 (1.4 – 2.7)	0.135
Fasting cortisol, nmol/l	169.0 (157.0 – 196.3)	170.5 (158.0 – 204.8)	172.5 (139.5 – 226.5)	198.0 (154.0 – 261.8)	0.900
Fasting GH, μg/l	0.3 (0.2 – 1.0)	0.3 (0.3 – 0.5)	0.2 (0.1 – 0.4)	0.2 (0.1 – 0.2)	0.096
Fasting PP, pmol/l	8.3 (4.4 – 13.6)	10.5 (6.5 – 17.1)	5.5 (5.0 – 8.1)	9.9 (5.9 – 14.9)	0.809
Fasting GLP-1, pmol/l	3.0 (2.4 – 3.6)	2.5 (1.5 – 3.5)	1.5 (1.0 – 2.1)	1.7 (1.5 – 3.1)	0.219
Fasting *R*_a_tot, mg kg^−1^ min^−1^	1.85 (1.60 – 2.11)	1.82 (1.74 – 1.92)	1.76 (1.71 – 2.01)	1.87 (1.56 – 2.09)	0.863
Fasting *R*_d_, mg kg^−1^ min^−1^	1.85 (1.60 – 2.11)	1.82 (1.74 – 1.92)	1.76 (1.71 – 2.01)	1.87 (1.56 – 2.09)	0.863
Fasting EGP, mg kg^−1^ min^−1^	1.82 (1.57 – 2.08)	1.79 (1.71 – 1.89)	1.73 (1.68 – 1.98)	1.84 (1.53 – 2.06)	0.863

Data are presented as median (IQR), unless otherwise specified

Hormones and glucose fluxes were assessed at the fasting state (t0)

^a^Calculated as the reciprocal of HOMA2 %S

In the fasting state (t0), blood glucose, endogenous insulin, C-peptide levels, insulin sensitivity and beta cell function did not differ significantly between the groups (all *p*>0.05), with all groups being insulin sensitive (HOMA-IR <1.0). No significant differences between groups were observed for the fasting levels of glucagon, adrenaline, noradrenaline, cortisol, GH, PP, GLP-1 and glucose fluxes (all *p*>0.05).

### Postprandial period

**Fig. 1:**
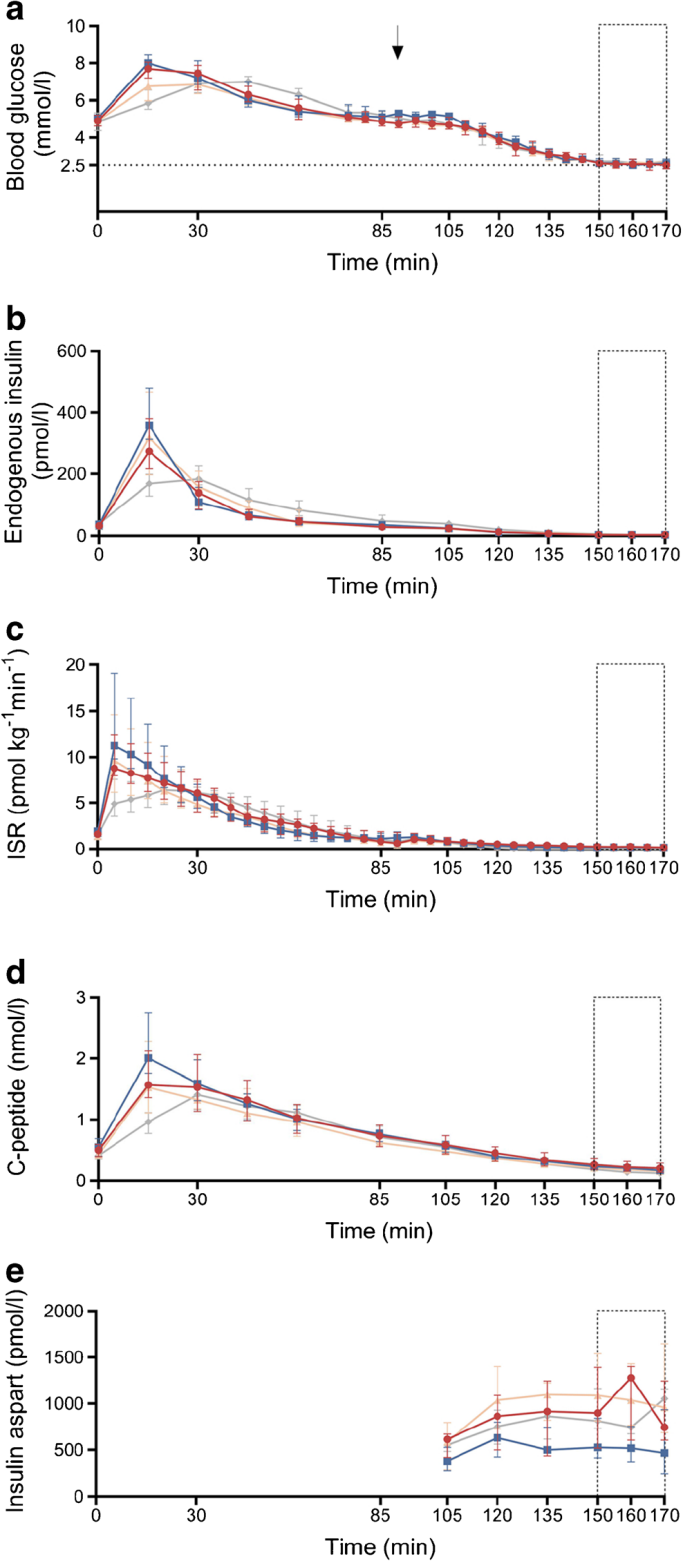
Profiles of blood glucose (**a**), endogenous insulin (**b**), ISR (**c**), C-peptide (**d**) and insulin aspart (**e**) over the entire experiment. Data are presented as median (IQR). The arrow shows the initiation of insulin aspart infusion (t90) and the rectangles with the dotted line correspond to the hypoglycaemic period (t150–t170). The horizontal dotted line in (**a**) represents the glucose target of 2.5 mmol/l during the hypoglycaemic period. Red circles, PBH group (*n*=8); blue squares, RYGB control group (*n*=8); orange triangles, SG control group (*n*=8); grey diamonds, CN (*n*=*8*)

Trajectories of blood glucose, endogenous insulin and ISR are displayed in Fig. 1a–c. A significant difference in the time-to-peak of the blood glucose was observed between the groups (*p*=0.035), with earlier peak levels in the RYGB control group vs CN (post hoc pairwise comparison: *p*=0.024). Peak blood glucose levels significantly differed between the groups (median [IQR]: PBH 7.9 [7.7–8.1] mmol/l; RYGB 8.4 [8.0–8.4] mmol/l; SG 7.1 [6.6–8.0] mmol/l; and CN 7.1 [6.8–7.5] mmol/l; *p*=0.038) but the post hoc analysis showed no significant pairwise differences. Time-to-peak of the endogenous insulin level significantly differed between the groups (*p*=0.001), with an earlier increase in both RYGB groups vs CN (both post hoc pairwise comparisons: *p*=0.007). Peak endogenous insulin concentrations were significantly higher in the RYGB control group vs CN (median [IQR]: RYGB 358.5 [315.5–421.7] pmol/l vs CN 210.0 [171.3–242.3] pmol/l; post hoc pairwise comparison, *p*=0.041). Peak ISR was also significantly higher in the RYGB control group than in CN (median [IQR]: RYGB 11.3 [10.4–15.8] pmol kg^−1^ min^−1^ vs CN 6.7 [5.8–7.2] pmol kg^−1^ min^−1^; post hoc pairwise comparison, *p*=0.008). However, ISR and insulin sensitivity did not significantly differ between the groups in the entire postprandial period, t0–t85 (*p*=0.656 and *p*=0.289, respectively) (ESM, Table 4). The total insulin clearance at t0–t85 was higher in the PBH group than in the SG control group (median [IQR]: 44.0 [41.0–51.5] ml kg^−1^ min^−1^ vs 34.7 [31.8–38.5] ml kg^−1^ min^−1^, respectively; post hoc pairwise comparison, *p*=0.043) (ESM, Table 4).

**Fig. 2:**
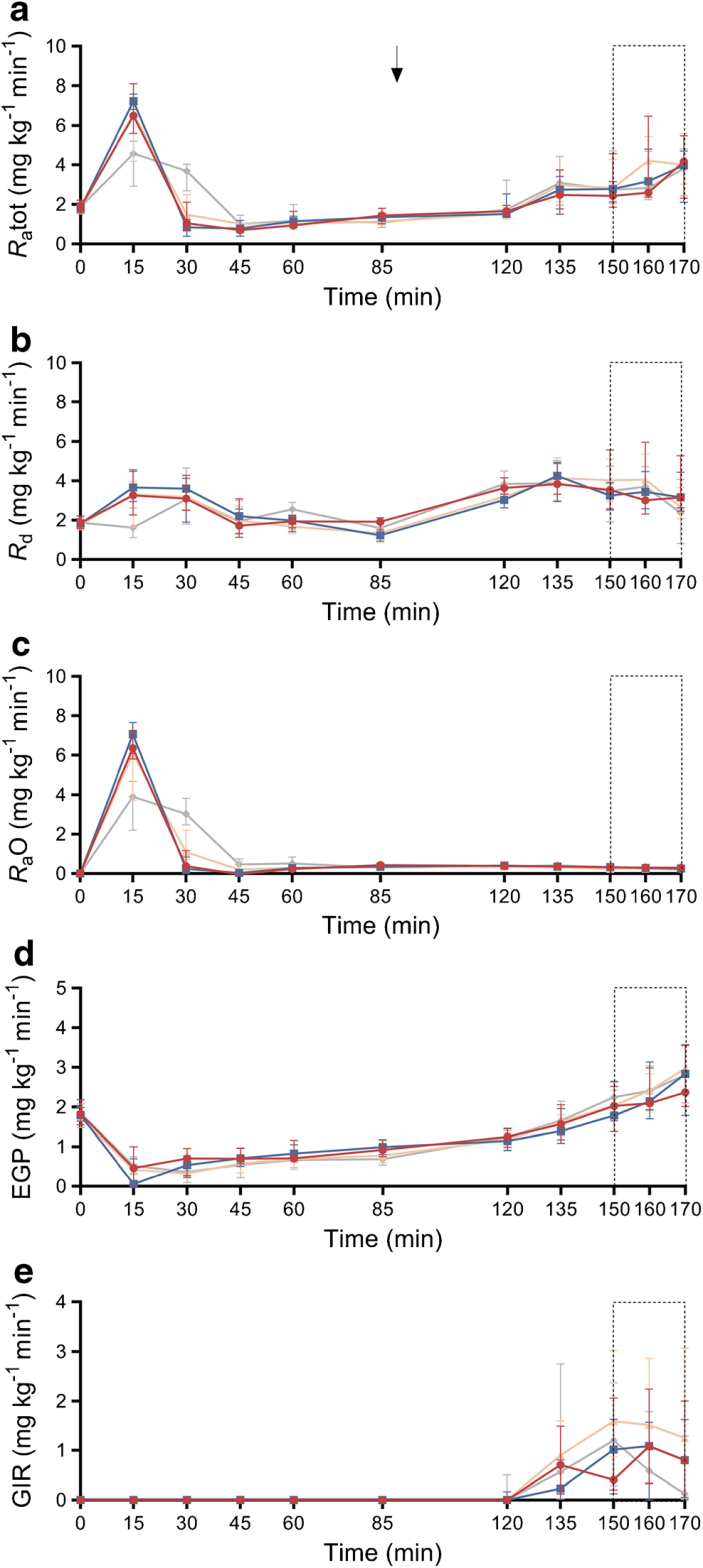
*R*_a_tot (**a**), *R*_d_ (**b**), *R*_a_O (**c**), EGP (**d**) and GIR (**e**) over the entire experiment. Data are presented as median (IQR). The arrow shows the initiation of insulin aspart infusion (t90) and the rectangles with the dotted line correspond to the hypoglycaemic period (t150–t170). Red circles, PBH group (*n*=8); blue squares, RYGB control group (*n*=8); orange triangles, SG control group (*n*=8); grey diamonds, CN (*n*=8)

Trajectories of glucose fluxes over the entire experiment are shown in Fig. 2a–d and ESM Table 4. At t15, *R*_a_O was significantly higher in both RYGB groups vs CN (both post hoc pairwise comparisons: *p*<0.01). The same was observed for *R*_a_tot and *R*_d_. EGP decreased across all groups at t15 (*p*<0.001) and the degree of EGP suppression did not differ significantly between the groups (*p*=0.841). The recovery, over 85 min, of glucose after oral administration was not significantly different between the groups (median [IQR]: PBH 62.9 [57.5–66.8]%; RYGB 63.0 [56.6–65.5]%; SG 63.9 [61.3–69.4]%; CN 65.0 [60.7–69.2]%; *p*=0.713).

### Hypoglycaemic period

The median (IQR) of the mean blood glucose during the hypoglycaemic period (t150–t170) was 2.6 (2.5–2.6) mmol/l in the PBH group, 2.6 (2.5–2.7) mmol/l in the RYGB control group, 2.6 (2.5–2.7) mmol/l in the SG control group and 2.7 (2.6–2.7) mmol/l in CN (Fig. 1a). The median (IQR) CV of the clamped blood glucose (SD divided by the mean blood glucose) during t150–t170 was 6.6 (4.1–7.8)% in the PBH group, 3.9 (3.2–4.6)% in the RYGB control group, 5.5 (3.2–6.9)% in the SG control group and 4.3 (3.6–6.2)% in CN (*p*=0.431). The mean endogenous insulin during t150-t170 was consistently low, without significant group differences (median [IQR]: PBH 3.0 [3.0–4.1] pmol/l; RYGB 3.0 [3.0–3.3] pmol/l; SG 3.5 [3.0–4.4] pmol/l; CN 4.2 [3.0–5.1] pmol/l; *p*=0.411), while C-peptide levels remained measurable (Fig. 1b,d). The median (IQR) of the mean insulin aspart levels during t150–t170 was 945.2 (671.5–1239.2) pmol/l in the PBH group, 520.2 (399.1–752.2) pmol/l in the RYGB control group, 1033.1 (964.0–1435.2) pmol/l in the SG control group and 881.8 (740.6–1171.5) pmol/l in CN (*p*=0.067) (Fig. 1e).

#### Hormonal response to hypoglycaemia

**Fig. 3:**
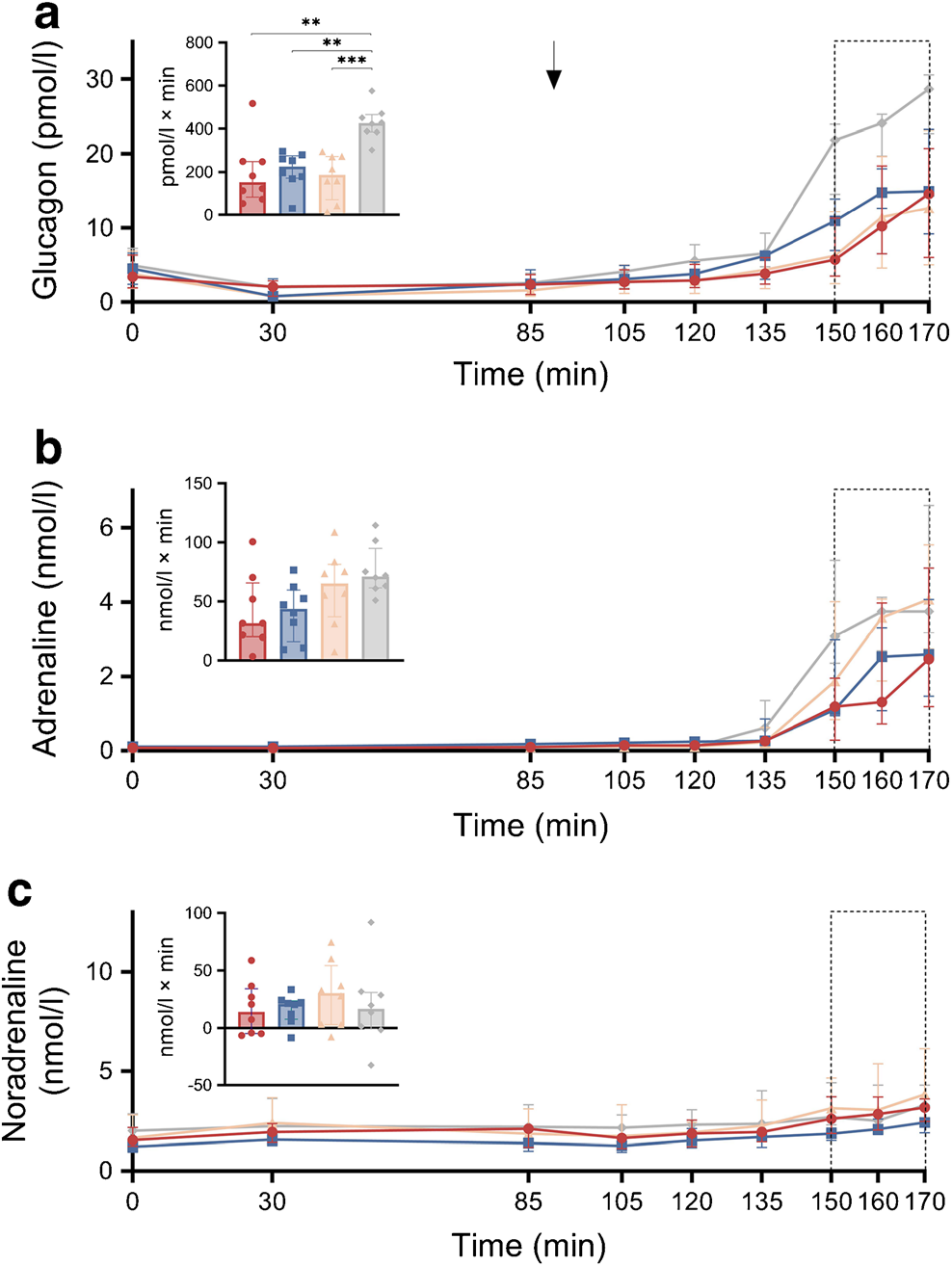
Profiles of glucagon (**a**), adrenaline (**b**) and noradrenaline (**c**) over the entire experiment. Data are presented as median (IQR). The arrow shows the initiation of insulin aspart infusion (t90) and the rectangles with the dotted line correspond to the hypoglycaemic period (t150–t170). The bar charts indicate the iAUC of the respective hormone during t150–t170 (relative to t85) and show the individual data as well as the median values (top of the bar) and the IQR (whiskers). Red circles, PBH group (*n*=8); blue squares, RYGB control group (*n*=8); orange triangles, SG control group (*n*=8); grey diamonds, CN (*n*=8). **p<0.01 and ***p<0.001 in the post hoc analysis

**Fig. 4:**
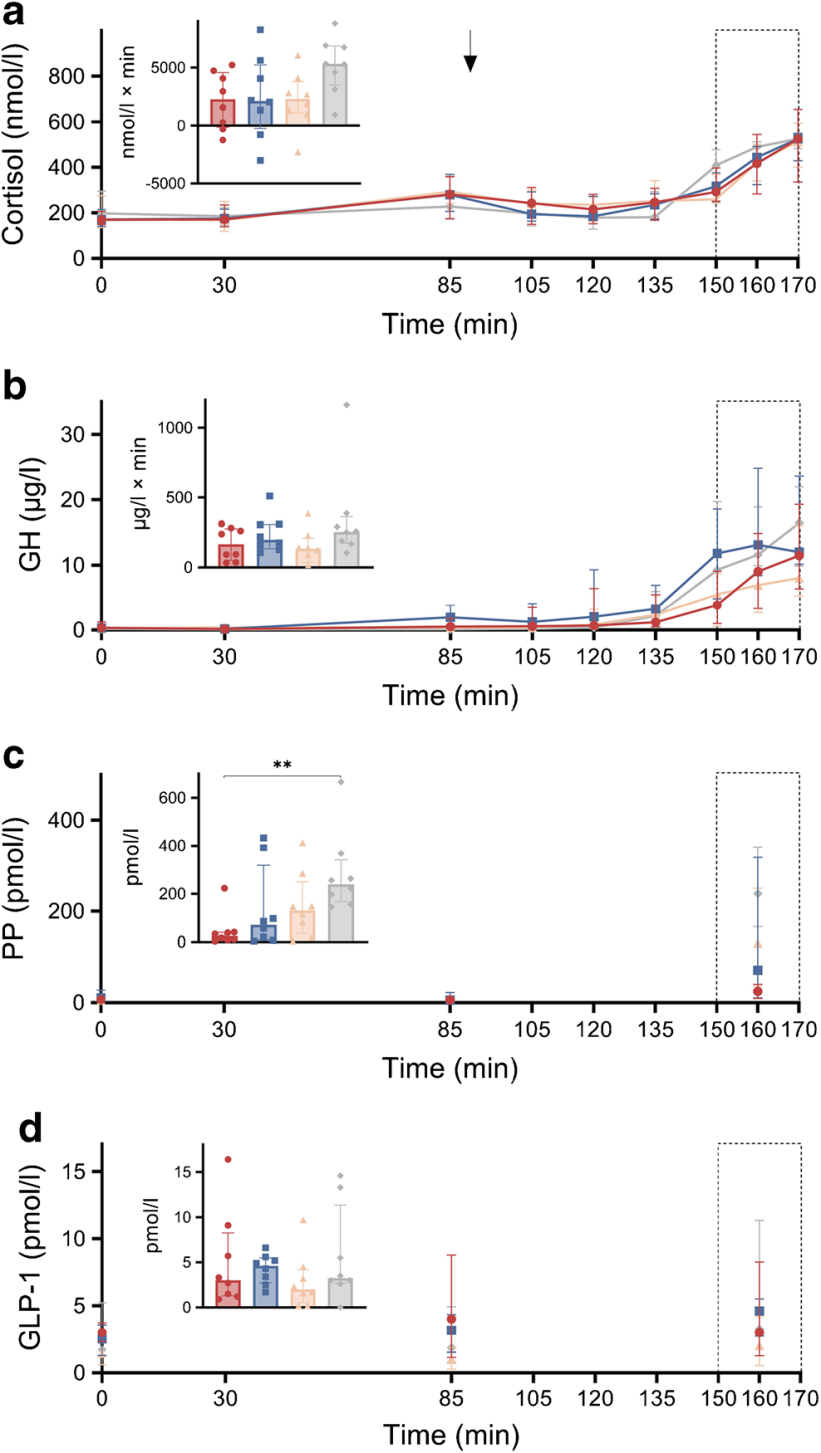
Profiles of cortisol (**a**), GH (**b**), PP (**c**) and GLP-1 (**d**) over the entire experiment. Data are presented as median (IQR). The arrow shows the initiation of insulin aspart infusion (t90) and the rectangles with the dotted line correspond to the hypoglycaemic period (t150–t170). The bar charts in (**a**) and (**b**) indicate the iAUC of the respective hormone during t150–t170 (relative to t85). The bar charts in (**c**) and (**d**) indicate the levels of the respective hormone at t160. PP and GLP-1 were only measured at t0, t85 and t160. The bar charts show the individual data as well as the median values (top of the bar) and the IQR (whiskers). Red circles, PBH group (*n*=8); blue squares, RYGB control group (*n*=8); orange triangles, SG control group (*n*=8); grey diamonds, CN (*n*=8). **p<0.01 in the post hoc analysis

Counter-regulatory hormones and GLP-1 levels during hypoglycaemia are shown in Figs 3 and 4 and ESM Table 5. All counter-regulatory hormones increased consistently during hypoglycaemia across all groups (all *p*<0.05). The glucagon response was lower in all surgical groups vs CN (all iAUC *p*<0.01). No significant differences were observed in other pairwise comparisons. The adrenaline response to hypoglycaemia was statistically different between the groups (iAUC *p*=0.045), but the post hoc analysis showed no significant pairwise differences (all *p*>0.05). Noradrenaline, cortisol and GH did not differ significantly between the groups (all iAUC *p*>0.05). Of note, the total PP concentration was significantly lower in the PBH group vs CN (median [IQR]: PBH 24.7 [10.9–38.7] pmol/l vs CN 238.7 [186.3–288.9] pmol/l; post hoc pairwise comparison, *p*=0.005). GLP-1 levels did not differ significantly between the groups (*p*=0.425).

#### Metabolic response to hypoglycaemia

Glucose fluxes during hypoglycaemia are shown in Fig. 2 and ESM Table 6. *R*_a_O was suppressed across all groups (*p*<0.001), while *R*_a_tot and *R*_d_ increased compared with t85 (both *p*<0.001), without significant group differences. EGP increased during hypoglycaemia across all groups (*p*<0.001) but did not significantly differ between the groups during t150–t170 (AUC *p*=0.471). The GIR was low overall (per-group median amount of infused glucose was 0.8–1.7 g during t150–t170), without significant group differences (AUC *p*=0.763).

Insulin sensitivity did not differ significantly between the groups during t150–t170 (*p*=0.131). Post-hepatic insulin clearance was significantly higher in the RYGB control group vs the SG control group and CN during hypoglycaemia (post hoc pairwise comparisons, *p*=0.041 and *p*=0.033, respectively) (ESM Table 6).

#### Cardiocirculatory response to hypoglycaemia

Heart rate and BP responses to hypoglycaemia are shown in ESM Table 7 and ESM Fig. 3. Heart rate increased across all groups (*p*<0.001) and did not significantly differ between the groups during hypoglycaemia (*p*=0.836). Systolic BP remained unchanged (*p*=0.637), whereas diastolic BP decreased across all groups during hypoglycaemia (*p*<0.001), without significant group differences (*p*=0.890 and *p*=0.817, respectively).

#### Hypoglycaemic symptoms

**Fig. 5:**
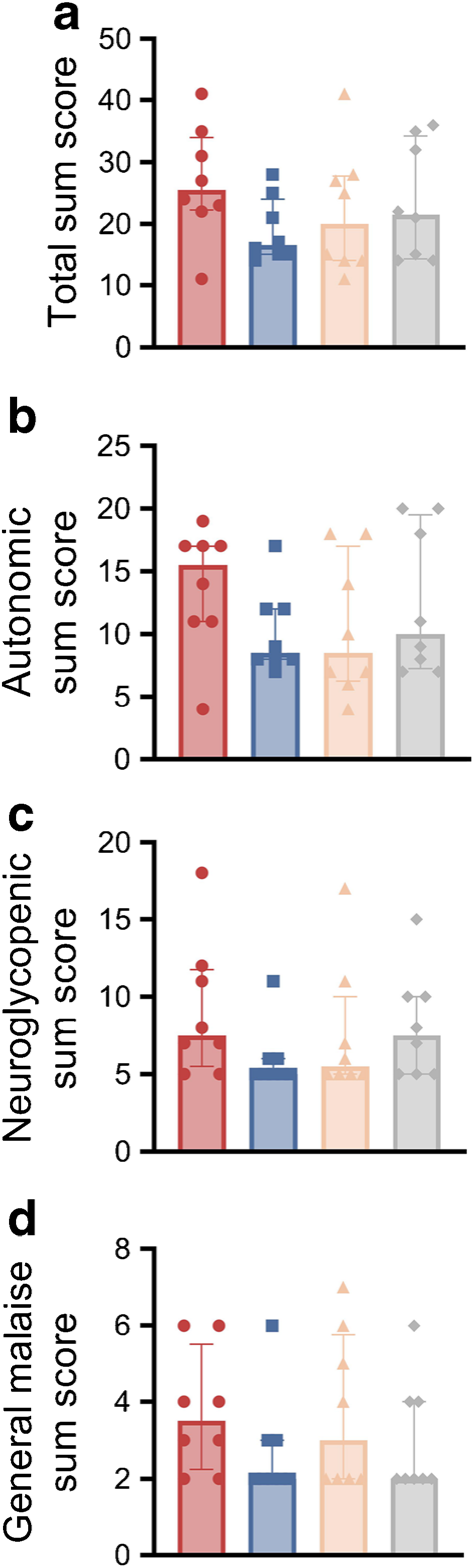
Total (**a**), autonomic (**b**), neuroglycopenic (**c**) and general malaise (**d**) sum scores during hypoglycaemia. The bar charts show the individual data as well as the median values (top of the bar) and the IQR (whiskers). Symptoms were assessed with the Edinburgh Hypoglycaemia Symptom Scale. The symptom sum score ranges are as follows: total sum score 11–77; autonomic sum score 4–28; neuroglycopenic sum score 5–35; and general malaise sum score 2–14. Red circles, PBH group (*n*=8); blue squares, RYGB control group (*n*=8); orange triangles, SG control group (*n*=8); grey diamonds, CN (*n*=8)

The symptom sum scores are shown in Fig. 5 and ESM Table 8. The total sum score was in the low-medium range and did not significantly differ between the groups (median [IQR]: PBH 25.5 [22.8–32.0]; RYGB 16.5 [15.0–22.0]; SG 20.0 [14.0–27.3]; CN 21.5 [14.8–32.8]; *p*=0.348). Across all groups, participants reported a median of 39.3% and 17.1% of the maximum intensity of the autonomic and neuroglycopenic sum scores, respectively.

## Discussion

In this study, we examined counter-regulatory hormones and glucose fluxes in a postprandial hypoglycaemic clamp experiment in patients with PBH after RYGB and surgical (RYGB, SG) and non-surgical control groups. We found reduced glucagon responses to hypoglycaemia in all surgical groups compared with CN. However, glucagon responses did not distinguish surgical groups with and without PBH. All other counter-regulatory hormonal responses did not differ significantly between the groups, apart from PP, which was significantly lower in the PBH group vs CN. Despite the different glucagon exposures, no significant differences in EGP were observed between the groups during hypoglycaemia. This finding is supported by available evidence suggesting that hepatic glucose production is regulated with redundancy at various levels [33].

The observed decreased glucagon exposure to hypoglycaemia after bariatric surgery is concordant with previous longitudinal studies suggesting that surgery-induced weight loss lowers the glucagon response to induced hypoglycaemia [34–36]. Decreased glucagon responses to insulin-induced hypoglycaemia were also demonstrated after short-term fasting (72 h) in healthy men [37]. These findings indicate that an experienced energy deficit, independently of its inductor (surgical or non-surgical), alters glucagon responses to hypoglycaemia. Although not statistically significant, a similar lower response to hypoglycaemia was observed for the second-most important counter-regulatory hormone, adrenaline, in the surgical groups vs CN. Likewise, PP, a surrogate marker of parasympathetic input to the pancreas and a marker (additional to glucagon and catecholamines) of autonomic hypoglycaemia counter-regulation [38], appeared to be lower in the surgical groups vs CN, with significant differences only between the PBH group and CN. Our findings are in line with pre-existing literature [34, 35, 36] and we speculate that they may suggest a global attenuation of neurohormonal responses to insulin-induced hypoglycaemia in post-bariatric surgery patients experiencing substantial weight loss. Of note, a recent study assessing brain glucose utilisation, blood flow and neuronal activity during euglycaemic and hypoglycaemic–hyperinsulinaemic clamps pre- and post-RYGB, found that attenuated counter-regulatory hormone responses post-surgery were accompanied by adaptive changes in the brain [36]. RYGB and SG re-route gastrointestinal anatomy and thus produce consistently lower postprandial nadir glucose levels [39, 40]. This may lead to decreased sensitivity to the level of hypoglycaemia and promote the use of alternative substrates, thereby deviating the need for prompt secretion of counter-regulatory hormones during hypoglycaemia [41]. Due to no significant group differences in cardiocirculatory responses and perceived autonomic symptoms, our findings were less indicative of an autonomic nervous system dysfunction. Of note, the tendency towards higher symptom scores in the patients with PBH could be interpreted as a facilitated symptom perception because they also experience symptoms in their daily life. Further, the absence of differences in GLP-1 exposure during hypoglycaemia does not support a contribution of GLP-1 towards the attenuated glucagon response in the surgical groups. Somatostatin, a potent inhibitor of both insulin and glucagon release, was not quantified in this study but a few human studies assessing somatostatin levels ≥1 year after bariatric surgery reported no surgery-induced changes [42, 43]. However, we cannot exclude the possibility that differences in intra-islet crosstalk and non-characterised gut-factors may contribute to the distinct secretory profiles of glucagon and PP [44, 45].

Against our hypothesis and indications from previous work using provocative tests [46], counter-regulatory responses to hypoglycaemia did not differ significantly between the PBH and the RYGB control group. Although attenuated counter-regulation may predispose to PBH, these response patterns may be rather characteristic of the altered post-bariatric surgery physiology (sustained weight loss and lower nadir glucose levels) rather than a root cause of PBH. Conversely, other pathophysiological factors, such as accelerated *R*_a_O, insulinotropic factors and greater postprandial insulin secretion have distinguished post-bariatric surgery individuals with and without PBH [11, 47]. Of note, no significant differences were observed between PBH patients and surgical control individuals in the early postprandial period. However, the comparably small oral glucose load (15 g) may have not been sufficient to unravel distinct patterns in the enteropancreatic axis and early postprandial glucose fluxes. Altogether, the high variability of clinical manifestations (from mild to severe phenotypes) supports a multifactorial model of glucose dysregulation in PBH.

The novelty of this study lies in the conductance of a standardised postprandial insulin-induced hypoglycaemic clamp experiment contrasting counter-regulatory responses in patients with confirmed symptomatic PBH after RYGB vs surgical and non-surgical control individuals with similar blood glucose and insulin concentrations. We acknowledge the small sample size, which may have led to type II errors and may not be entirely representative of the PBH, RYGB and SG populations. However, the groups were comparable in terms of age, sex, body composition, HbA_1c_, fasting hormonal concentrations and insulin sensitivity. Furthermore, we acknowledge that a longitudinal design could control for intra- and inter-individual variability, although such a study design would be hardly feasible due to the unknown number of post-surgery individuals developing PBH and the remarkably long follow-up (>1year) required to avoid interferences with early post-operative dynamics. Due to the predominantly postprandial occurrence of PBH and the well-established differences in counter-regulatory responses to insulin-induced hypoglycaemia in the fasting vs postprandial state [48], we performed the clamp experiment after the ingestion of 15 g of glucose. The small oral glucose load was chosen to ensure complete absorption by the time of hypoglycaemia. The control algorithm for GIR adjustment allowed us to reach target hypoglycaemia following standardised glucose trajectories. Still, we acknowledge that the hypoglycaemia was induced under experimental and not real-life conditions, when insulin concentrations would be lower. However, due to the comparably low insulin infusion rate (two to three times lower compared with other studies) [30, 34, 35], the influence on counter-regulation may be limited [49]. Finally, a prolonged hypoglycaemia might have revealed additional findings but such protocols might have raised ethical challenges due to potential safety concerns.

In conclusion, the glucagon response to standardised, insulin-induced postprandial hypoglycaemia is attenuated after both RYGB and SG vs CN. These findings support adaptive reactions to bariatric surgery that can predispose to PBH. However, glucagon and other counter-regulatory responses did not significantly differ between symptomatic PBH patients and surgical control individuals. Therefore, we conclude that attenuated counter-regulation is not a root cause of PBH but may be a contributor to its multifactorial and complex aetiology.

## Supplementary Information

ESM (PDF 1.08 kb)

## Data Availability

The dataset generated and analysed during the current study is available from the corresponding author upon reasonable request.

## Abbreviations

CN: Non-surgical control group
EGP: Endogenous glucose production
GH: Growth hormone
GIR: Glucose infusion rate
GLP-1: Glucagon-like peptide-1
iAUC: Incremental AUC
ISR: Insulin secretion rate
PBH: Post-bariatric hypoglycaemia
PP: Pancreatic polypeptide
*R*_a_O: Rate of glucose appearance after oral administration
*R*_a_tot: Rate of total glucose appearance
*R*_d_: Rate of glucose disappearance
RYGB: Roux-en-Y gastric bypass
SG: Sleeve gastrectomy
