# Supplementary material for: Counter-regulatory responses to postprandial hypoglycaemia in patients with post-bariatric hypoglycaemia vs surgical and non-surgical control individuals
Source: Diabetologia. 2023 Jan 17;66(4):741–53. doi: 10.1007/s00125-022-05861-9 (PMC9947092; doi:10.1007/s00125-022-05861-9)
Supplement: Supplementary file 1 — (PDF 1.08 kb) [file 125_2022_5861_MOESM1_ESM.pdf]

Electronic Supplementary Material

Content

Methods 2
Calculation of the glucose fluxes 2
Calculation of the post-hepatic insulin clearance 4
ESM Table 1: Characterisation of the PBH group 5
ESM Table 2: Type and time points of the examined variables 6
ESM Table 3: Precision details of the used assays 7
ESM Table 4: Postprandial glucose fluxes, recovery of oral glucose and insulin metrics 8
ESM Table 5: Hormonal response to hypoglycaemia 9
ESM Table 7: Cardiocirculatory response to hypoglycaemia 11
ESM Table 8: Hypoglycaemic symptoms 12
ESM Fig. 1: Overview of the experimental flow 13
ESM Fig. 2: Flow diagram 14
ESM Fig. 3: Cardiocirculatory profiles 15
References 16

Methods

Calculation of the glucose fluxes

For calculating the glucose fluxes, tracer and tracee plasma glucose concentrations were firstly derived from plasma mass percent excess (MPE) and total glucose concentration data (G^{tot}) [1-3].

In particular, assuming that both the [6,6-^{2}H2] and [U-^{13}C]glucose tracers are almost absent in natural glucose, the [6,6-^{2}H2]glucose tracer concentration in plasma (G^{2H2}) was obtained as:

$$G^{2H2}(t) = \frac{MPE^{2H2}(t)}{\left(1 - MPE^{2H2}(t)\right) \cdot purity^{2H2} + MPE^{2H2}(t)} \cdot \left(G^{tot}(t) - G^{U13C}(t)\right) \qquad \text{Eq. (1)}$$

assuming that the [U-^{13}C]glucose tracer does not contribute to the MPE^{2H2} measurement and that $purity^{2H2}$ is the purity of the [6,6-$^2H_2$]glucose tracer in the infusate (here equal to 98%).

Similarly, the [U-^{13}C]glucose tracer concentration in plasma (G^{U13C}) was obtained as:

$$G^{U13C}(t) = \frac{MPE^{U13C}(t)}{\left(1 - MPE^{U13C}(t)\right) \cdot purity^{U13C} + MPE^{U13C}(t)} \cdot G^{tot}(t) \qquad \text{Eq. (2)}$$

assuming that [6,6-$^2H_2$] contributes like natural glucose to the MPE^{U13C} measurement and $purity^{U13C}$ is the purity of the [U-^{13}C]glucose tracer in the ingested glucose (here equal to 99%).

Finally, the endogenous glucose concentration (G^{end}) was calculated as:

$$G^{end}(t) = G^{tot}(t) - G^{2H2}(t) - G^{U13C}(t) \cdot \left(1 + {}^{1}/_{TTR_{meal}}\right) \qquad \text{Eq. (3)}$$

where TTR_{meal} is the tracer-to-tracee ratio in the ingested glucose.

Glucose fluxes (the rate of glucose appearance after oral administration [R_aO], the endogenous glucose production [EGP], the rate of total glucose appearance [$R_a$tot] and the rate of glucose disappearance [R_d]) were then calculated from a dual-tracer approach [4] using an one-compartment model of glucose kinetics [5]. In particular, the unknown R_aO was obtained as:

$$RaO(t) = \left[\frac{Inf^{2H2}(t)}{\frac{G^{2H2}(t)}{G^{U13C}(t)}} - \frac{p \cdot V \cdot G^{U13C}(t)}{\frac{G^{2H2}(t)}{G^{U13C}(t)}} \cdot \frac{d\left(\frac{G^{2H2}(t)}{G^{U13C}(t)}\right)}{dt}\right] \cdot \left(1 + {}^{1}/_{TTR_{meal}}\right) \qquad \text{Eq. (4)}$$

where Inf^{2H2} is the rate of infusion of the [6,6-$^2H_2$]glucose tracer, p is the pool fraction and V is the total volume of distribution (here fixed to 0.65 and 200 ml/kg, respectively [3]).

Similarly, the EGP was calculated as:

$$EGP(t) = \frac{Inf^{2H2}(t)}{\frac{G^{2H2}(t)}{G^{end}(t)}} - \frac{p \cdot V \cdot G^{end}(t)}{\frac{G^{2H2}(t)}{G^{end}(t)}} \cdot \frac{d\left(\frac{G^{2H2}(t)}{G^{end}(t)}\right)}{dt} \qquad \text{Eq. (5)}$$

Combining R_aO and EGP, together with Inf^{2H2} and the glucose infusion rate (GIR) administered during the hypoglycaemic clamp, the $R_a$tot was obtained as:

$$Ratot(t) = RaO(t) + EGP(t) + Inf^{2H2}(t) + GIR(t) \qquad \text{Eq. (6)}$$

Finally, the R_d was calculated as:

$$Rd(t) = Ratot(t) - p \cdot V \cdot \frac{d(G^{tot}(t))}{dt} \qquad \text{Eq. (7)}$$

Calculation of the post-hepatic insulin clearance

Post-hepatic insulin clearance (CL) was calculated from the insulin aspart infusion (Inf^I) during the hypoglycaemic clamp. Specifically, a linear one-compartment model of insulin kinetics was assumed [6], so that:

$$V_I \cdot \dot{I}(t) = -CL \quad \cdot I(t) + Inf^I(t) \qquad \text{Eq. (8)}$$

where V_I is the volume of insulin distribution and I the insulin aspart concentration measured by LC-MS/MS. The model was identified on insulin aspart data, assuming measurement error to be independent, Gaussian, with zero mean and constant SD estimated a posteriori [7].

ESM Table 1: Characterisation of the PBH group

	PBH group (*n*=8)
History of severe hypoglycaemia, *n* (%)[a]	3 (37.5%)
Time since diagnosis, years	4.0 (1.8–5.3)
PBH treatment, *n* (%)	
Patients with pharmacotherapy at enrolment (acarbose)[b]	1 (12.5%)
Patients with a history of previous pharmacotherapy	4 (50.0%)
Acarbose	4 (50.0%)
Diazoxide	2 (25.0%)
Sodium-glucose Co-transporter-2 inhibitor	1 (12.5%)
Patients with previous invasive interventions due to PBH	4 (50.0%)
Dietary modification	6 (75.0%)
Frequency of symptoms, *n* (%)	
More than one per day	2 (25.0%)
More than one per week	3 (37.5%)
More than one per month	1 (12.5%)
More than one per trimester	1 (12.5%)
Less than one per trimester	1 (12.5%)
Type of symptoms	
Autonomic, *n* (%)	
Perspiration	7 (87.5%)
Tremor	5 (62.5%)
Palpitations	2 (25.0%)
Hunger	3 (37.5%)
Neuroglycopenic, *n* (%)	
Loss of concentration	5 (62.5%)
Incoordination	1 (12.5%)
Visual disturbances	4 (50.0%)
Dizziness	1 (12.5%)
General Malaise, *n* (%)	
Nausea	1 (12.5%)
Fatigue	1 (12.5%)

Data are presented as *n* (percentage) or median (IQR)
[a]Defined as an event associated with severe cognitive impairment (including loss of consciousness and seizures) requiring external assistance by another person. Information was collected by the medical records.
[b]Stopped prior to study-related procedures

ESM Table 2: Type and time points of the examined variables

Time point	-100	0	15	25	30	45	55	60	75	80	85	90	95	100	105	110	115	120	125	130	135	140	145	150	155	160	165	170
Blood glucose	✓	✓	✓		✓	✓		✓	✓	✓	✓	✓	✓	✓	✓	✓	✓	✓	✓	✓	✓	✓	✓	✓	✓	✓	✓	✓
Endogenous insulin		✓	✓		✓	✓		✓			✓				✓			✓			✓			✓		✓		✓
C-peptide		✓	✓		✓	✓		✓			✓				✓			✓			✓			✓		✓		✓
Insulin aspart															✓			✓			✓			✓		✓		✓
Glucagon		✓			✓						✓				✓			✓			✓			✓		✓		✓
Catecho-lamines		✓			✓						✓				✓			✓			✓			✓		✓		✓
Cortisol		✓			✓						✓				✓			✓			✓			✓		✓		✓
GH		✓			✓						✓				✓			✓			✓			✓		✓		✓
PP		✓									✓															✓		
GLP-1		✓									✓															✓		
Isotopic glucose enrichments	✓	✓	✓		✓	✓		✓			✓							✓			✓			✓		✓		✓
BP		✓		✓			✓			✓						✓				✓				✓			✓	
Edinburgh Hypoglycaemia Symptom Scale		✓		✓			✓			✓							✓			✓				✓			✓	
ECG														✓														

ESM Table 3: Precision details of the used assays

Parameter	Type of assay	Intra-assay CV (%)	Inter-assay CV (%)
Endogenous insulin[8]	Immunoassay	0.8–4.3	1.4–5.3
C-peptide[9]	Immunoassay	1.7–2.3	2.9–4.8
Insulin aspart[10]	LC-MS/MS	6.1–8.4	-
Glucagon[11]	Immunoassay	2.1–14.0	7.0–16.0
Adrenaline[12]	LC-MS/MS	3.0–9.5	5.4–18.8
Noradrenaline[12]	LC-MS/MS	6.0–18.8	6.5–13.4
Cortisol[13]	Immunoassay	1.1–5.5	1.8–7.3
GH[14]	Immunoassay	2.9–4.6	4.2–6.6
PP1-36[15]	LC-MS/MS	1.0–15.0	10.0–21.0
PP3-36[15]	LC-MS/MS	4.0–17.0	9.0–11.0
GLP-1[16]	Immunoassay	4.5–5.5	7.1–10.9
[6,6-$^{2}H_2$]glucose[17][a]	GC-CI/MS	0.2–0.4	0.8–3.5
[U-^{13}C]glucose[17][a]	GC/C/IRMS	0.4	0.7

[a]Data for Relative Standard Deviations are shown

ESM Table 4: Postprandial glucose fluxes, recovery of oral glucose and insulin metrics

	PBH group (*n*=8)	RYGB control group (*n*=8)	SG control group (*n*=8)	CN (*n*=8)	*p* value
AUC$_{0\text{-}15}$ of $R_a$tot, mg/kg	60.7 (58.5–73.0)**	68.5 (65.7–70.2)***	62.3 (48.3–65.1)	50.0 (34.0–53.4)	<0.001
AUC$_{0\text{-}15}$ of R_d, mg/kg	36.3 (31.7–47.1)*	40.3 (35.3–48.4)*	39.9 (27.2–42.7)	27.1 (20.4–30.5)	0.018
AUC$_{0\text{-}15}$ of R_aO, mg/kg	47.7 (43.7–52.2)**	53.0 (47.4–54.8)***	45.6 (32.8–50.5)	29.3 (17.4–32.2)	<0.001
Suppression of EGP (t15), %	-73.6 ((-86.2)–(-55.6))	-96.8 ((-106.3)–(-69.2))	-75.4 ((-97.2)–(-66.4))	-69.4 ((-83.1)–(-57.0))	0.841
Recovery of oral glucose (t0–t85), %	62.9 (57.5–66.8)	63.0 (56.6–65.5)	63.9 (61.3–69.4)	65.0 (60.7–69.2)	0.713
AUC$_{0\text{-}85}$ of ISR, pmol/kg	396.4 (326.6–506.6)	379.9 (341.3–478.9)	317.0 (290.1–448.3)	369.4 (311.4–408.2)	0.656
Insulin sensitivity (t0–t85), ml kg^{-1} min^{-1} per nmol/l	2.4 (2.0–3.0)	2.2 (1.7–2.3)	1.9 (1.7–2.1)	1.7 (1.7–2.5)	0.289
Total insulin clearance (t0–t85), ml kg^{-1} min^{-1}	44.0 (41.0–51.5)†	40.3 (36.7–42.5)	34.7 (31.8–38.5)	31.9 (29.4–41.1)	0.026

Data are presented as median (IQR)
**p* <0.05 vs CN in the post hoc analysis
***p* <0.01 vs CN in the post hoc analysis
****p* <0.001 vs CN in the post hoc analysis
†*p* <0.05 vs the SG control group in the post hoc analysis

ESM Table 5: Hormonal response to hypoglycaemia

		PBH group (*n*=8)	RYGB control group (*n*=8)	SG control group (*n*=8)	CN (*n*=8)	*p* value
Glucagon	$Mean_{150\text{-}170}$, pmol/l	10.4 (6.4–15.3)*	13.8 (11.7–15.7)*	10.9 (6.9–16.7)**	24.1 (21.5–26.0)	0.005
	$iAUC_{150\text{-}170}$, pmol/l × min	152.9 (103.7–248.7)**	225.5 (176.7–266.2)**	186.7 (129.4–271.5)***	425.6 (387.8–456.0)	<0.001
Adrenaline	$Mean_{150\text{-}170}$, nmol/l	1.8 (1.1–2.7)	2.4 (1.5–3.0)	3.4 (2.4–4.0)	3.3 (2.9–4.2)	0.126
	$iAUC_{150\text{-}170}$, nmol/l × min	31.6 (21.1–56.5)	43.7 (26.9–54.8)	65.2 (49.1–77.4)	71.1 (62.6–81.8)	0.045
Noradre-naline	$Mean_{150\text{-}170}$, nmol/l	2.9 (2.1–3.6)	2.4 (2.0–2.5)	3.3 (2.3–5.0)	2.9 (2.4–3.5)	0.192
	$iAUC_{150\text{-}170}$, nmol/l × min	14.1 ((-4.4)–29.3)	20.9 (10.7–22.7)	30.3 (3.3–42.6)	16.6 (0.7–29.4)	0.767
Cortisol	$Mean_{150\text{-}170}$, nmol/l	408.2 (303.3–488.8)	435.0 (347.2–457.3)	394.3 (359.6–466.9)	475.8 (445.2–494.0)	0.481
	$iAUC_{150\text{-}170}$, nmol/l × min	2260.0 (150.0–4237.5)	2107.5 (810.0–4451.3)	2292.5 (1280.0–3138.8)	5315.0 (4226.3–6792.5)	0.099
GH	$Mean_{150\text{-}170}$, µg/l	9.6 (4.2–14.4)	12.0 (9.6–19.6)	7.0 (4.4–9.0)	12.9 (9.8–16.3)	0.090
	$iAUC_{150\text{-}170}$, µg/l × min	166.8 (78.7–266.6)	198.1 (148.7–306.3)	134.1 (74.4–164.4)	254.7 (185.4–315.0)	0.174
PP	Levels at t160, pmol/l	24.7 (10.9–38.7)**	71.0 (17.0–170.6)	130.6 (66.4–181.4)	238.7 (186.3–288.9)	0.009
GLP-1	Levels at t160, pmol/l	3.0 (1.4–6.6)	4.6 (3.2–5.3)	2.0 (1.3–3.5)	3.3 (2.9–7.5)	0.425

Data are presented as median (IQR)
**p* <0.05 vs CN in the post hoc analysis
***p* <0.01 vs CN in the post hoc analysis
****p* <0.001 vs CN in the post hoc analysis

ESM Table 6: Metabolic response to hypoglycaemia

		PBH group (*n*=8)	RYGB control group (*n*=8)	SG control group (*n*=8)	CN (*n*=8)	*p* value
Total glucose appearance	Mean rate$_{150\text{-}170}$, mg kg^{-1} min^{-1}	2.83 (2.34–5.63)	3.41 (2.20–3.94)	3.81 (3.27–4.28)	3.18 (2.39–4.48)	0.959
	AUC$_{150\text{-}170}$, mg/kg	55.41 (46.95–116.90)	67.00 (44.77–81.39)	76.98 (63.62–89.84)	75.63 (47.66–96.01)	0.971
Glucose disappearance	Mean rate$_{150\text{-}170}$, mg kg^{-1} min^{-1}	3.12 (2.60–5.66)	3.35 (2.46–3.90)	3.67 (2.82–4.16)	2.96 (2.33–3.88)	0.900
	AUC$_{150\text{-}170}$, mg/kg	61.60 (50.34–114.51)	69.05 (49.81–77.70)	75.63 (60.16–85.36)	69.05 (50.22–96.88)	0.954
Glucose appearance after oral administration	Mean rate$_{150\text{-}170}$, mg kg^{-1} min^{-1}	0.31 (0.27–0.34)	0.28 (0.26–0.36)	0.24 (0.21–0.31)	0.26 (0.22–0.30)	0.532
	AUC$_{150\text{-}170}$, mg/kg	6.12 (5.46–6.72)	5.62 (5.19–7.20)	4.83 (4.14–6.22)	5.13 (4.41–7.36)	0.670
EGP	Mean rate$_{150\text{-}170}$, mg kg^{-1} min^{-1}	2.13 (1.99–2.60)	2.21 (1.91–2.80)	2.43 (2.28–2.84)	2.49 (2.14–2.82)	0.806
	AUC$_{150\text{-}170}$, mg/kg	42.37 (39.92–52.24)	43.86 (38.23–55.71)	48.55 (45.52–56.02)	51.08 (42.75–70.05)	0.471
Glucose infusion	Mean rate$_{150\text{-}170}$, mg kg^{-1} min^{-1}	0.69 (0.31–2.0)	0.95 (0.26–1.38)	1.47 (0.78–2.58)	0.63 (0.33–1.45)	0.766
	AUC$_{150\text{-}170}$, mg/kg	15.78 (7.04–41.04)	18.12 (6.47–28.34)	30.60 (15.80–48.35)	13.09 (7.69–29.27)	0.763
Insulin sensitivity[a]	t150–t170, ml kg^{-1} min^{-1} per nmol/l	0.98 (0.74–1.26)	1.32 (0.91–1.69)	0.83 (0.72–1.11)	0.80 (0.55–0.94)	0.131
Post-hepatic insulin clearance[a]	t150–t170, ml kg^{-1} min^{-1}	6.73 (4.89–9.24)	12.20 (8.25–14.60)*	5.83 (4.01–6.48)	5.82 (4.19–7.97)	0.024

Data are presented as median (IQR)
[a]*n*=7 in the CN
**p* <0.05 vs the SG control group and CN in the post hoc analysis

ESM Table 7: Cardiocirculatory response to hypoglycaemia

		PBH group (*n*=8)	RYGB control group (*n*=8)	SG control group (*n*=8)	CN (*n*=8)	*p* value
Heart rate[a]	$Mean_{150\text{-}170}$, bpm	73.6 (65.7–79.9)	64.4 (62.0–72.5)	70.8 (65.2–80.5)	72.5 (69.1–77.2)	0.836
	Difference to t0, bpm	8.5 (5.7–10.7)	5.7 ((-1.8)–12.4)	10.9 (3.4–14.7)	3.5 (0.8–8.0)	0.560
Systolic BP	Levels at t165, mmHg	115.0 (110.5–129.5)	126.0 (112.5–131.8)	120.0 (111.0–133.0)	119.0 (113.8–125.3)	0.890
	Difference to t0, mmHg	2.5 ((-1.3)–5.8)	0.5 ((-2.3)–3.5)	0.0 ((-11.0)–6.0)	-5.5 ((-13.0)–2.0)	0.766
Diastolic BP	Levels at t165, mmHg	67.0 (62.5–70.8)	71.0 (56.8–75.3)	68.5 (61.5–76.0)	68.5 (57.8–72.0)	0.817
	Difference to t0, mmHg	-7.5 ((-8.3)–(-6.5))	-10.5 ((-12.5)–(-3.5))	-10.5 ((-15.5)–(-2.5))	-13.5 ((-14.3)–(-9.0))	0.167

Data are presented as median (IQR)
[a]*n*=7 in the CN

ESM Table 8: Hypoglycaemic symptoms

	PBH group (*n*=8)	RYGB control group (*n*=8)	SG control group (*n*=8)	CN (*n*=8)	*p* value
Total sum score	25.5 (22.8–32.0)	16.5 (15.0–22.0)	20.0 (14.0–27.3)	21.5 (14.8–32.8)	0.348
Autonomic sum score	15.5 (11.0–17.0)	8.5 (8.0–12.0)	8.5 (6.8–15.0)	10.0 (7.8–18.5)	0.436
Neuroglycopenic sum score	7.5 (5.0–11.3)	5.0 (5.0–6.0)	5.5 (5.0–8.0)	7.5 (5.0–10.0)	0.447
General malaise sum score	3.5 (2.8–6.0)	2.0 (2.0–3.0)	3.0 (2.0–5.3)	2.0 (2.0–4.0)	0.357

Data are presented as median (IQR)

ESM Fig. 1: Overview of the experimental flow

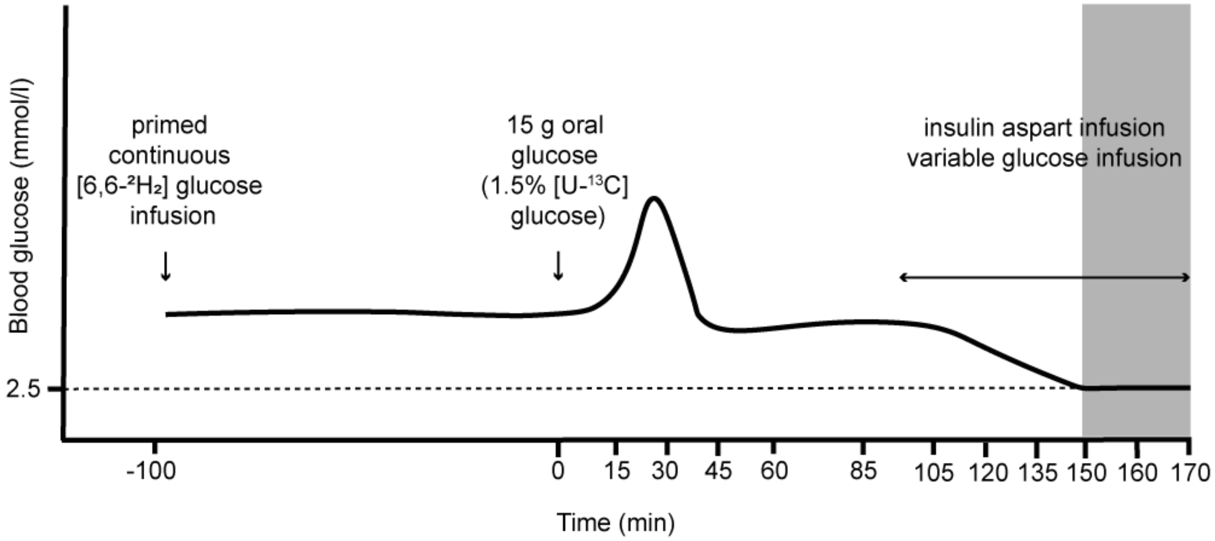

ESM Fig. 2: Flow diagram

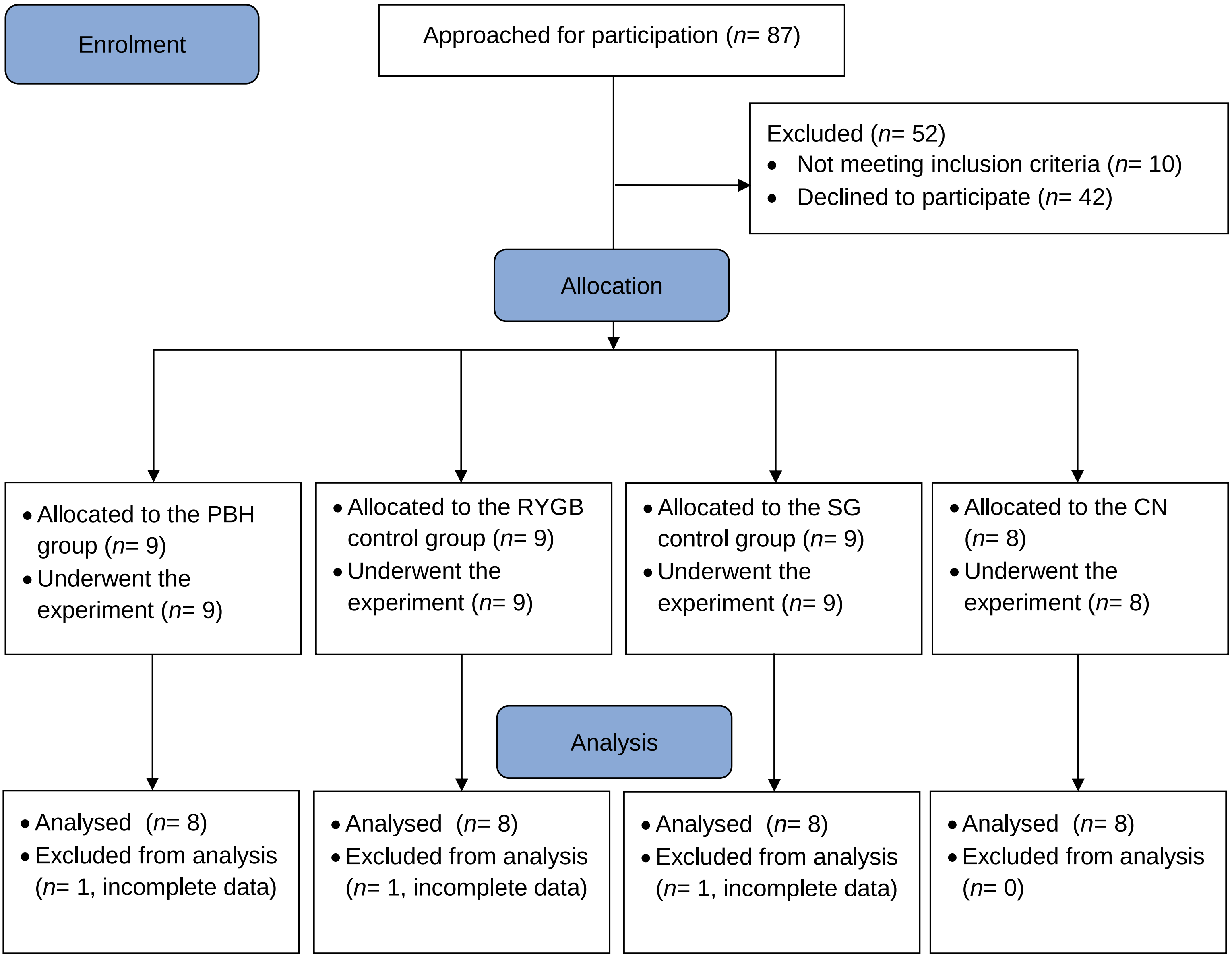

ESM Fig. 3: Cardiocirculatory profiles

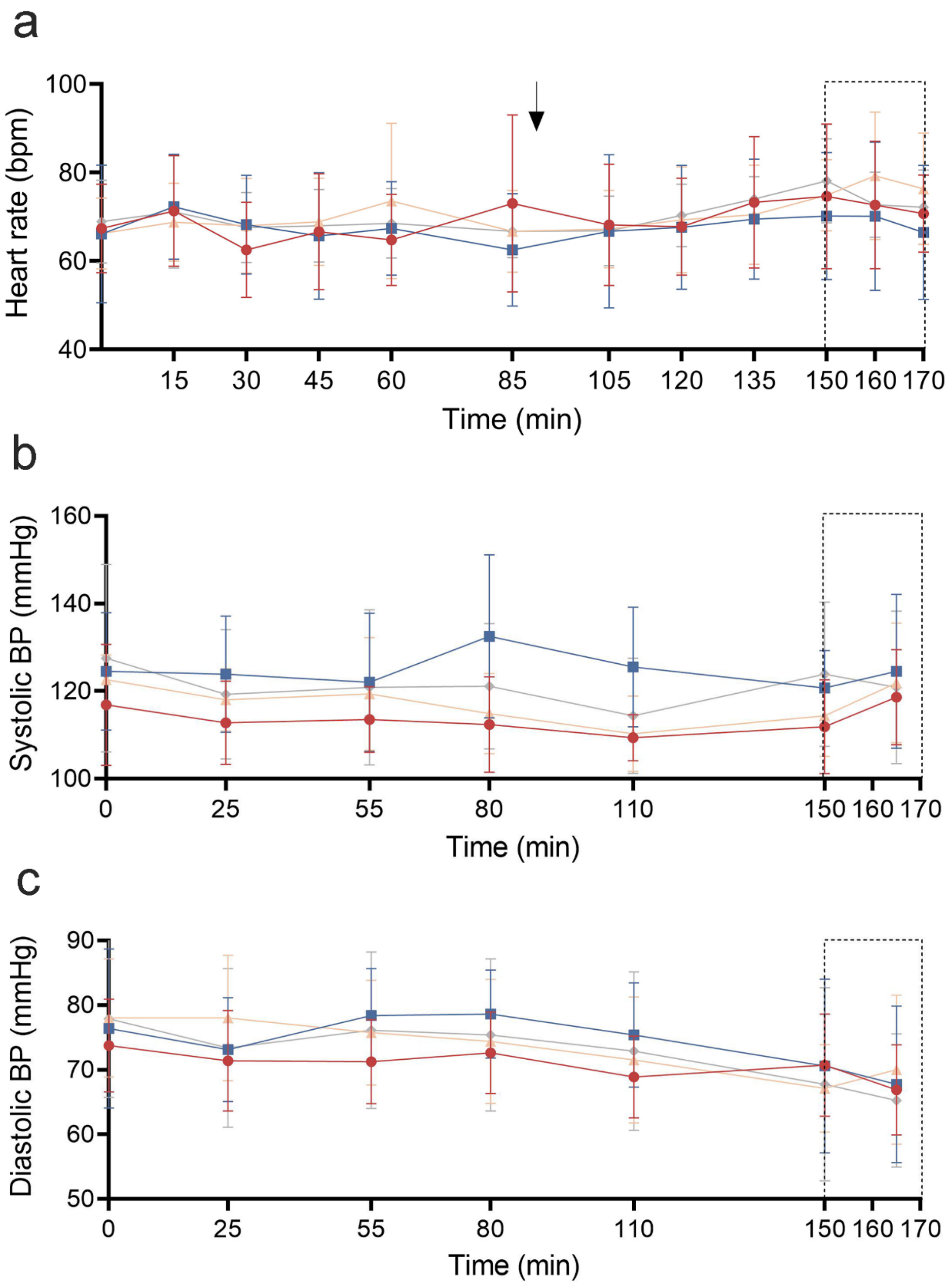

Profiles of heart rate (a), systolic BP (b) and diastolic BP (c) over the entire experiment. Data are presented as median (IQR). The arrow shows the initiation of insulin aspart infusion (t90) and the rectangles with the dotted line correspond to the hypoglycaemic period (t150–t170). Red circles, PBH group (n=8); blue squares, RYGB control group (n=8); orange triangles, SG control group (n=8); grey diamonds, CN (n=8 for BP and n=7 for heart rate)

References

[1] Cobelli C, Toffolo G, Foster DM (1992) Tracer-to-tracee ratio for analysis of stable isotope tracer data: link with radioactive kinetic formalism. Am J Physiol 262(6 Pt 1): E968-975. 10.1152/ajpendo.1992.262.6.E968

[2] Moran A, Toffolo G, Schiavon M, et al. (2018) A novel triple-tracer approach to assess postprandial protein turnover. Am J Physiol Endocrinol Metab 315(4): E469-e477. 10.1152/ajpendo.00012.2018

[3] Basu R, Di Camillo B, Toffolo G, et al. (2003) Use of a novel triple-tracer approach to assess postprandial glucose metabolism. Am J Physiol Endocrinol Metab 284(1): E55-69. 10.1152/ajpendo.00190.2001

[4] Rizza RA, Toffolo G, Cobelli C (2016) Accurate Measurement of Postprandial Glucose Turnover: Why Is It Difficult and How Can It Be Done (Relatively) Simply? Diabetes 65(5): 1133-1145. 10.2337/db15-1166

[5] Steele R, Bjerknes C, Rathgeb I, Altszuler N (1968) Glucose uptake and production during the oral glucose tolerance test. Diabetes 17(7): 415-421. 10.2337/diab.17.7.415

[6] Toffolo G, Campioni M, Basu R, Rizza RA, Cobelli C (2006) A minimal model of insulin secretion and kinetics to assess hepatic insulin extraction. Am J Physiol Endocrinol Metab 290(1): E169-e176. 10.1152/ajpendo.00473.2004

[7] Cobelli C, Carson E (2019) Introduction to Modeling in Physiology and Medicine. 2nd edn. Academic Press

[8] Roche (2021) Elecsys Insulin. Available from https://assets.cwp.roche.com/f/94122/x/4e3cccec43/insulin-factsheet.pdf. Accessed 08 November 2022

[9] Siemens (2018) Immulite 2000 C-Peptide. Available from https://zentrallaborweb.uniklinikum-saarland.de/labor/data/Beipackzettel/Immulite/CPEPS1/C-Peptide_-_IMMULITE_2000_Systems_-_Rev_09.pdf. Accessed 08 November 2022

[10] Thomas A, Yang R, Petring S, Bally L, Thevis M (2020) Simplified quantification of insulin, its synthetic analogs and C-peptide in human plasma by means of LC-HRMS. Drug Test Anal 12(3): 382-390. 10.1002/dta.2765

[11] Mercodia (2020) Update of the Mercodia Glucagon ELISA. Available from https://www.mercodia.com/update-of-the-mercodia-glucagon-elisa/. Accessed 01 May 2022 2022

[12] Dunand M, Gubian D, Stauffer M, Abid K, Grouzmann E (2013) High-throughput and sensitive quantitation of plasma catecholamines by ultraperformance liquid chromatography-tandem mass spectrometry using a solid phase microwell extraction plate. Anal Chem 85(7): 3539-3544. 10.1021/ac4004584

[13] Roche (2020) Elecsys Cortisol II. Available from http://193.191.178.147/Mithras/Analyses.nsf/ecbed47964dbd5b9c1256cc6003eb951/2e2fc7f37a4165a8c1256c22002fc16d/$FILE/Cortisol%20II-6-e8-122021.pdf. Accessed 08 November 2022

[14] Siemens (2018) Immulite 2000 Growth Hormone (hGH). Available from https://www.yumpu.com/es/document/view/18931491/immuliter-2000-growth-hormone-hgh. Accessed 08 November 2022

[15] Reverter-Branchat G, Eugster PJ, Kuenzli C, et al. (2022) Multiplexed Assay to Quantify the PP-Fold Family of Peptides in Human Plasma Using Microflow Liquid Chromatography–Tandem Mass Spectrometry. Clinical Chemistry 68(4): 584-594. 10.1093/clinchem/hvab229

[16] Immuno-Biological Laboratories Co. L (2018) GLP-1, Active form Assay Kit - IBL. Available from https://www.ibl-japan.co.jp/files/topics/1699_ext_02_en_0.pdf. Accessed 08 November 2022

[17] Sauvinet V, Gabert L, Qin D, Louche-Pélissier C, Laville M, Désage M (2009) Validation of pentaacetylaldononitrile derivative for dual 2H gas chromatography/mass spectrometry and 13C gas chromatography/combustion/isotope ratio mass spectrometry analysis of glucose. Rapid Commun Mass Spectrom 23(23): 3855-3867. 10.1002/rcm.4294
